# Membrane Active Peptides Remove Surface Adsorbed Protein Corona From Extracellular Vesicles of Red Blood Cells

**DOI:** 10.3389/fchem.2020.00703

**Published:** 2020-08-11

**Authors:** Priyanka Singh, Imola Cs. Szigyártó, Maria Ricci, Ferenc Zsila, Tünde Juhász, Judith Mihály, Szilvia Bősze, Éva Bulyáki, József Kardos, Diána Kitka, Zoltán Varga, Tamás Beke-Somfai

**Affiliations:** ^1^Institute of Materials and Environmental Chemistry, Research Centre for Natural Sciences, Budapest, Hungary; ^2^MTA-ELTE Research Group of Peptide Chemistry, Eötvös Loránd University, Budapest, Hungary; ^3^Department of Biochemistry, Institute of Biology, Eötvös Loránd University, Budapest, Hungary

**Keywords:** extracellular vesicles, antimicrobial peptide, liposome, biomembrane, protein corona

## Abstract

Besides the outstanding potential in biomedical applications, extracellular vesicles (EVs) are also promising candidates to expand our knowledge on interactions between vesicular surface proteins and small-molecules which exert biomembrane-related functions. Here we provide mechanistic details on interactions between membrane active peptides with antimicrobial effect (MAPs) and red blood cell derived EVs (REVs) and we demonstrate that they have the capacity to remove members of the protein corona from REVs even at lower than 5 μM concentrations. In case of REVs, the Soret-band arising from the membrane associated hemoglobins allowed to follow the detachment process by flow-Linear Dichroism (flow-LD). Further on, the significant change on the vesicle surfaces was confirmed by transmission electron microscopy (TEM). Since membrane active peptides, such as melittin have the affinity to disrupt vesicles, a combination of techniques, fluorescent antibody labeling, microfluidic resistive pulse sensing, and flow-LD were employed to distinguish between membrane destruction and surface protein detachment. The removal of protein corona members is a newly identified role for the investigated peptides, which indicates complexity of their *in vivo* function, but may also be exploited in synthetic and natural nanoparticle engineering. Furthermore, results also promote that EVs can be used as improved model systems for biophysical studies providing insight to areas with so far limited knowledge.

## Introduction

Biological membranes are constituted by a variable and complex mixture of lipids and proteins organized into a lipid bilayer structure (Sperelakis, 2012; Nickels et al., 2017). Besides membrane inserted proteins, *in vivo* systems can also contain significant amount of biomolecules adsorbed on their surface, also known as protein coronas (Nguyen and Lee, 2017). These protein layers may alter numerous membrane-related interactions and properties of natural systems, e.g., viral envelopes or extracellular vesicles (Monopoli et al., 2012; Bros et al., 2018; Buzás et al., 2018; Cai and Chen, 2019; Ezzat et al., 2019), but may also influence delivery or immunorecognition of synthetic nanoparticles (Oh et al., 2018). Since protein coronas form the first line in contact with other species, they are important both for the better mechanistic understanding of a diverse set of membrane active molecules, i.e., peptides, proteins, and also for understanding vesicular trafficking or viral fusion (Bros et al., 2018; Pattipeiluhu et al., 2020). However, compared to standard membrane models, our knowledge is very little on structural properties of biomembranes with an associated protein corona. Typical model membrane systems in structural biophysics are Langmuir monolayers, vesicles, liposomes, and solid supported lipid bilayers (Knobloch et al., 2015; Palivan et al., 2016), which in one hand provide systems that can be easily produced, but on the other hand may often limit the overall insight. Having an expanded set of complex membrane models is thus important to reach for advanced membrane biophysics (Stone and Deber, 2017; Rojalin et al., 2019). Accordingly, several directions are addressed, from preparation of more complex lipid bilayers by membrane protein reconstitution (Shen et al., 2013; Goers et al., 2018; Smirnova et al., 2018) to purified membranes from e.g., red blood cells (Li and Lykotrafitis, 2014; Rossi et al., 2019; Deák et al., 2020). Related, extracellular vesicles (EVs) may also offer new model systems to address problems described above. EVs have recently become an intensively studied area due to their extreme biomedical potential. These species also contain a phospholipid bilayer, with characteristic diameter ranging from 40 to 1000 nm and arising from diverse origin (Valadi et al., 2007; Vlassov et al., 2012; Zonneveld et al., 2014; Théry et al., 2018). Some of their subtypes may carry complex cargo, such as RNA (Keerthikumar et al., 2016; Kim et al., 2017; Driedonks and Nolte-'t Hoen, 2019), thus rendering them to play an important role in intercellular communication and potentially in diagnostics as biomarkers (Szabo and Momen-Heravi, 2017). Due to their *in vivo* origin, EVs also can contain adsorbed proteins and protein coronas (Rosa-Fernandes et al., 2017; Karasu et al., 2018; Xia et al., 2019). One of the frequently used EVs are red blood cell derived EVs (REVs), which offer a reproducible system with high particle concentration (Szigyártó et al., 2018; Kitka et al., 2019; Deák et al., 2020; Varga et al., 2020).

To progress in use of EVs as improved model membrane systems, here we employed red blood cell derived EVs and two selected membrane active peptides (MAPs) to characterize their interaction network utilizing several biophysical methods. Among membrane active compounds, antimicrobial peptides (AMPs) receive considerable interest as they have the potential to combat e.g., resistant bacterial species (Bradshaw, 2003). Natural AMPs, or host defense peptides, are components of the innate immune system, with a short sequence (~10–40 amino acids) and high content of cationic and hydrophobic residues. Although AMPs have multiple intracellular targets, the majority of their action mechanisms are based on the lysis of membranes (Brogden, 2005; Pasupuleti et al., 2012; Haney et al., 2019). Their *in vivo* structure-function relationship, however, is still not well-understood, which can be also due to the major differences between synthetic liposomes and natural biomembranes. For current studies the natural peptide melittin and its synthetic derivative, CM15, was selected as these are well-characterized with several of their membrane-related functions and hemolytic activities understood. Melittin is the major component of honey bee venom with potent lytic activity, as well as with multiple pharmacological actions e.g., antibacterial, antiviral, anticancer, and antiinflammatory effects (Dosler et al., 2016; Liu et al., 2016; Uddin et al., 2016). Its membrane binding mechanism can vary, either acting by a carpet mechanism, adsorbing on by bilayers in a partial helix conformation (Ohki et al., 1994) or forming toroidal pores (Smith et al., 1994; Sengupta et al., 2008; Lee et al., 2013; Hur et al., 2017) which probably requires self-assembly to tetrameric forms (Kurgan et al., 2019). Similarly to melittin, for the cecropin-melittin hybrid CM15, both carpet mechanism (Pistolesi et al., 2007) and pore formation were observed in a concentration dependent manner (Milani et al., 2009), but, in contrast to melittin, without significant hemolytic activity. To study the interactions between REV membranes and these MAPs, we performed complex biophysical characterization using linear dichroism (LD), circular dichroism (CD), attenuated total reflection Fourier-transform infrared spectroscopy (ATR-FTIR), dynamic light scattering (DLS), microfluidic resistive pulse sensing measurements (MRPS), fluorescent labeling coupled to size exclusion chromatography, and freeze-fracture transmission electron microscopy (FF-TEM). Current results indicate several novel functions of the investigated MAPs, including eradication of surface proteins from REVs upon their interaction.

## Materials and Methods

### Red Blood Cell Derived EVs (REVs) Isolation

15 mL blood was collected from healthy adult volunteers with informed consent in tripotassium ethylenediaminetetraacetic acid containing tubes (K3EDTA, Greiner Bio-One). The use of human blood samples was approved by the Scientific and Research Ethics Committee of the Hungarian Medical Research Council (ETT TUKEB 6449-2/2015) and during all procedures we followed the guidelines and regulations of the Helsinki Declaration from 1975. REV isolation was performed by a differential centrifugation protocol. Briefly, the red blood cells (RBCs) were isolated by centrifugation at 2500 × g for 10 min at 4°C (Nüve NF 800R centrifuge). To achieve complete removal of platelets and buffy coat, RBCs were washed with physiological salt solution three times at 2500 × g 10 min at 4°C. Buffy coat free red blood cells were suspended in PBS, and were kept 7 days at 4°C for vesicle production. The cells and the cellular debris were removed by centrifugation steps at 2500 × g for 15 min and 3000 × g for 30 min at room temperature. The supernatant containing REVs was further centrifuged at 16,000 × g for 30 min at 4°C (Eppendorf 5415R, F45-24-11 rotor). The REV pellets were suspended in PBS, and stored at 4°C until further use.

For spectroscopic experiments the REV sample was further purified, with size-exclusion chromatography (SEC) using a 3.5 mL gravity column filled with Sepharose CL-2B gel (GE Healthcare, Sweden). Hundred microliters REV sample was pipetted onto the column followed by addition of 900 μl PBS while the flow through was discarded. The purified REV was eluted with PBS and collected in 1 mL.

### Polarized Light Spectroscopy

Linear dichroism (LD), a spectroscopic technique used with oriented systems, is defined as the difference in absorption of the light polarized parallel and perpendicular relative to the flow direction (Equation 1).

(1) $$\begin{array}{r} \text{LD}={\text{A}}_{\left|\right|}-A_{⊥} \end{array}$$

Coupled to a Couette flow cell, where the shear force is created by rotation of the inner cylinder, the lipid vesicles can be distorted and give information about orientation angles of the associated and/or inserted molecules to the bilayer (Rodger et al., 2002; Nordén et al., 2010; Jonsson et al., 2013; Kogan et al., 2014; Rocha et al., 2016). The degree of orientation can be derived from the orientation of membrane probes inserted into the lipid bilayer, in our case pyrene (Py), which possess transition dipole moments with well-defined directions. Pyrene has several electronic transitions, among them one is polarized parallel (340 nm) and one transition is perpendicular (275 nm) to the long axis of molecules. The efficiency of orientation can be described by the orientation factor *S* (Equation 2)

(2) $$\begin{array}{r} LD^{r}=\frac{LD}{A_{iso}}=\frac{3}{4}S\left(1-3cos^{2}\alpha \right) \end{array}$$

where LD^r^ is the reduced LD signal, A_iso_ is the isotropic absorption and α is the angle of the transition dipole moment of the chromophore relative to the membrane normal. Insertion angle relative to the REV membrane normal of pyrene was determined previously (Szigyártó et al., 2018).

LD spectra were recorded using a JASCO-1500 spectrometer equipped with a Couette flow cell system. The vesicles were oriented under a shear gradient of 2270 s^−1^ with a total path length of 0.5 mm. Baselines at zero shear gradient were measured and subtracted from all spectra.

The secondary structure of peptides was characterized by circular dichroism (CD) spectroscopy experiments. CD spectra were recorded without rotation on a Jasco J-715 spectropolarimeter in a 0.1 cm path length rectangular quartz cuvette (Hellma, USA), between 195 and 500 nm in 1 nm increments at a scan speed of 100 nm/min. CD curves were corrected by the spectral contribution of the blank buffer solution.

### Dynamic Light Scattering (DLS)

Average size and size distribution of the DOPC and DOPC/DOPG vesicles as reference sample and isolated REVs were measured by W130i dynamic light scattering instrument (DLS, AvidNano, UK). The data obtained was analyzed using i-Size 3.0 software.

### Attenuated Total Reflection Fourier Transform Infrared Spectroscopy (ATR-FTIR)

ATR-FTIR measurements were recorded on a Varian 2000 FTIR Scimitar spectrometer (Varian Inc., USA). The “Golden Gate” (Specac Ltd, UK) single reflection ATR accessory was used with liquid nitrogen cooled mercury-cadmium-telluride (MCT) detector. 5μL of sample was mounted on the diamond ATR surface and a thin dry film was obtained by slowly evaporation of the buffer under ambient conditions (~10 min). 64 scans at a nominal resolution of 2 cm^−1^ was used and ATR correction was performed after each spectra acquisition. The Origin 2018 software package (OriginLab Corporation, Northampton, MA, USA) was used for all spectra analysis.

### Freeze-Fracture Transmission Electron Microscopy (FF-TEM)

FF-TEM was used for the study of the EVs morphology, by rapid freezing of sample without using fixation or negative staining materials (Severs, 2007; Deák et al., 2015). Approximately 2 μl of the sample were pipetted onto a gold sample holders and frozen in liquid Freon at −194°C and then stored in liquid nitrogen. Fracturing was performed at −100°C in a Balzers freeze-fracture device (Balzers BAF 400D, Balzers AG, Liechtenstein). The replica of the fractured surfaces was made using carbon-platinum shadowing, cleaned, and washed with surfactant solution and distilled water. The replica was transferred to a 200 mesh copper grid. Measurements were performed in a JEOL JEM 1011 transmission electron microscope operating at 80 kV and images were taken with Olympus Morada 11-megapixel camera and iTEM software (Olympus).

### Microfluidic Resistive Pulse Sensing (MRPS)

MRPS was used to quantify the effect of melittin on the size-distribution and concentration of REVs. Resistive pulse sensing (RPS) is a technique based on the Coulter-principle for measuring the size and concentration of vesicles. Microfluidic realization of RPS with a built-in pre-filter and a fixed constriction size overcomes many limitations of classical RPS technique (Fraikin et al., 2011; Varga et al., 2018, 2020). MRPS measurements were performed with an nCS1 instrument (Spectradyne LLC, USA). The samples were diluted 10-fold with bovine serum albumin (BSA, Sigma-Aldrich, Hungary) solution at 1 mg/mL in PBS buffer (Sigma-Aldrich, Hungary), filtered through a VivaSpin 500 [100 kDa MWCO membrane filter (Sartorius, Germany)] according to the manufacturer's instructions. All measurements were performed using factory calibrated TS-400 cartridges with a measurement range from 65 to 400 nm.

### Size Exclusion Chromatography With On-Line Fluorescence Detection (Flu-SEC)

Flu-SEC was used to quantify the effect of melittin on the amount of REV-associated proteins (e.g., hemoglobin) and glycophorin A [a specific marker for REVs (Kitka et al., 2019)]. In SEC, the components of the sample are separated based on their size. REVs can be separated from soluble proteins such as plasma proteins and antibodies with a properly chosen stationary phase, e.g., with the Sepharose CL-2B cross-linked agarose gel (GE Healthcare Bio-Sciences AB, Sweden). 100 μL of purified REV sample (with or without the addition of melittin) was incubated with 1 μL anti-CD235a-PE for 30 min at 37°C. 10 μL of labeled REV sample was injected into a Jasco HPLC system (Jasco, Tokyo, Japan) consisting of a PU-2089 pump with a UV-2075 UV/Vis detector and a FP-2020 fluorescence detector controlled by the Chromnav software v. 1.17.02. Tricorn 5/200 glass columns (GE Healthcare Bio-Sciences AB, Sweden), filled with Sepharose CL-2B were used, and the eluent was PBS with a flow rate of 0.5 mL/min. Absorbance spectra was recorded at 414 nm which corresponds to the absorption band of hemoglobin, and the area under the curve (AUC) of the REV-peak was used to quantify the amount of hemoglobin. The fluorescence spectra were collected at excitation and emission wavelength corresponding to PE fluorochrome (565/578 nm), and the AUC of the REV-peak was used to quantify the loss of glycophorin A upon interaction of REVs with melittin.

## Results and Discussion

**Figure 1:**
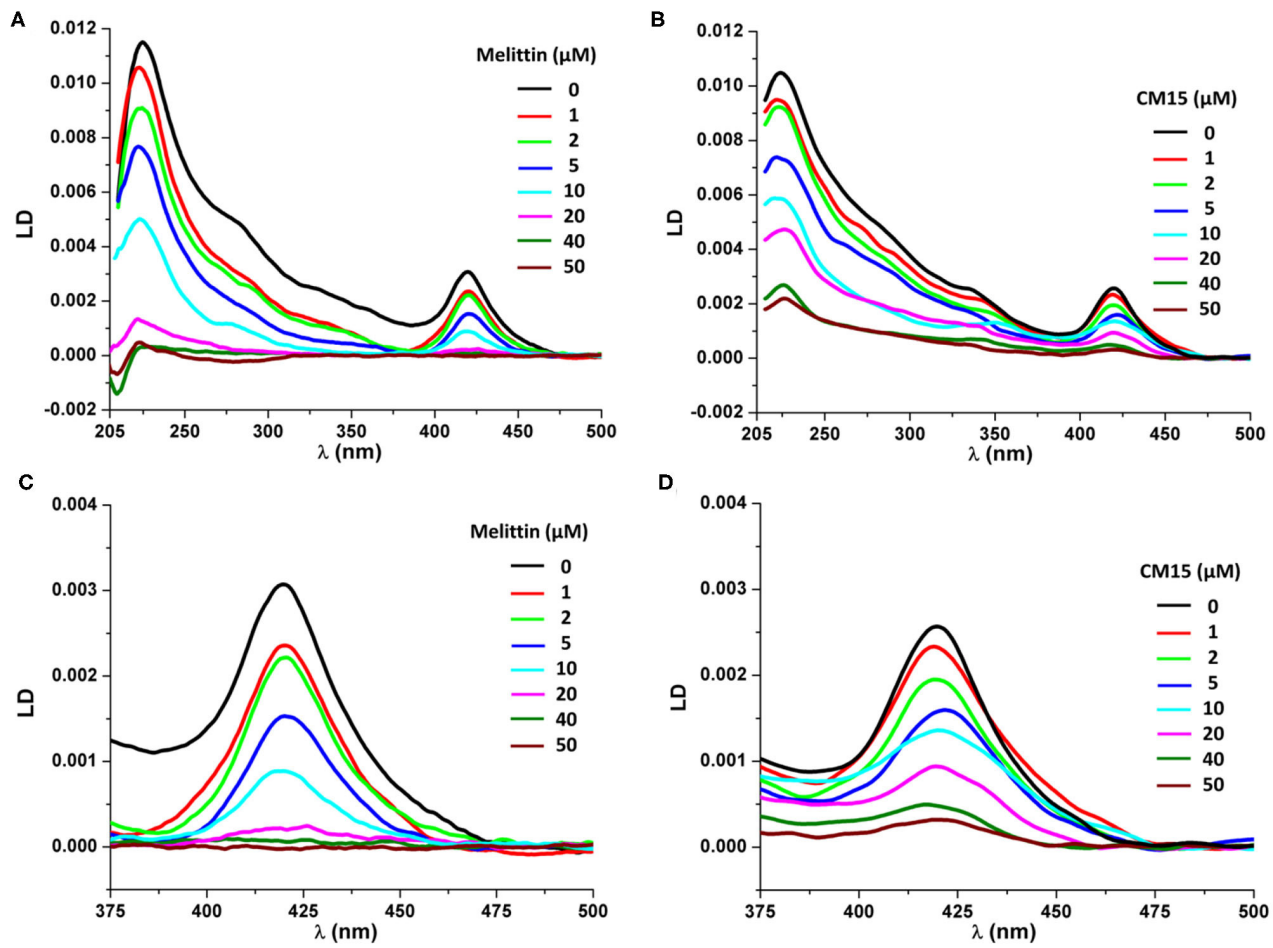
LD spectra of REVs in the presence of the investigated peptides. LD spectra of REVs upon addition of melittin **(A)** and CM15 **(B)**. Signal changes at the Soret band of REV samples in the function of melittin **(C)** and CM15 **(D)** concentration.

To understand the structural effect of membrane active peptides on REVs polarized light spectroscopy measurements were performed, using circular dichroism (CD) and flow-linear dichroism (LD) (Kogan et al., 2011, 2014; Rocha et al., 2016). Flow-LD spectroscopy is regularly used to study shear-aligned vesicles, where shear is created in a Couette flow cell, which measurements can provide both qualitative and quantitative details about membrane orientation and membrane associated molecules (Rodger et al., 2002; Nordén et al., 2010; Svensson et al., 2011). Furthermore, as reported earlier (Szigyártó et al., 2018) can be employed also to study molecular details of REV systems. The latter study has demonstrated that REVs contain membrane associated hemoglobins as part of their protein corona, which provides additional contributions to the LD spectra, owing to their heme groups (Caesar et al., 2009; Szigyártó et al., 2018). Considering LD measurements, the spectrum of control REV samples shows a major, positive band at ~220 nm, arising most probably from a mixture of the n-π^*^ and π-π^*^ amide transitions of helices present in REV proteins, where helices are predominantly aligned parallel to the macroscopic orientation (Figure 1). Note that due to the complex sample composition at this region significant light scattering occurs as well (Nordén et al., 2010). The spectrum also displays a positive peak at ~420 nm, which corresponds to the Soret band of heme containing proteins (Caesar et al., 2009; Szigyártó et al., 2018). In the case of REVs, only hemoglobin can be considered, thus the particular peak belongs to membrane associated hemoglobin molecules. The gradual increase in peptide concentrations up to 50 μM, shows the decrease of both positive peaks (Figure 1). In case of melittin, the peak at 420 nm shows a decrease even at 1 μM (Figure 1C), whereas for CM15 the decrease is slower (Figure 1D). Moreover, upon further addition of melittin and CM15, a small red and blue shift can be detected, respectively and shoulders emerge at higher wavelengths. The deconvoluted LD band (Szigyártó et al., 2018) shows components close to 417 and 430 nm, corresponding likely to a mixture of oxygen-bound (oxyHb) and oxygen-free (deoxyHb) hemoglobin (van Kampen and Zijlstra, 1983).

**Figure 2:**
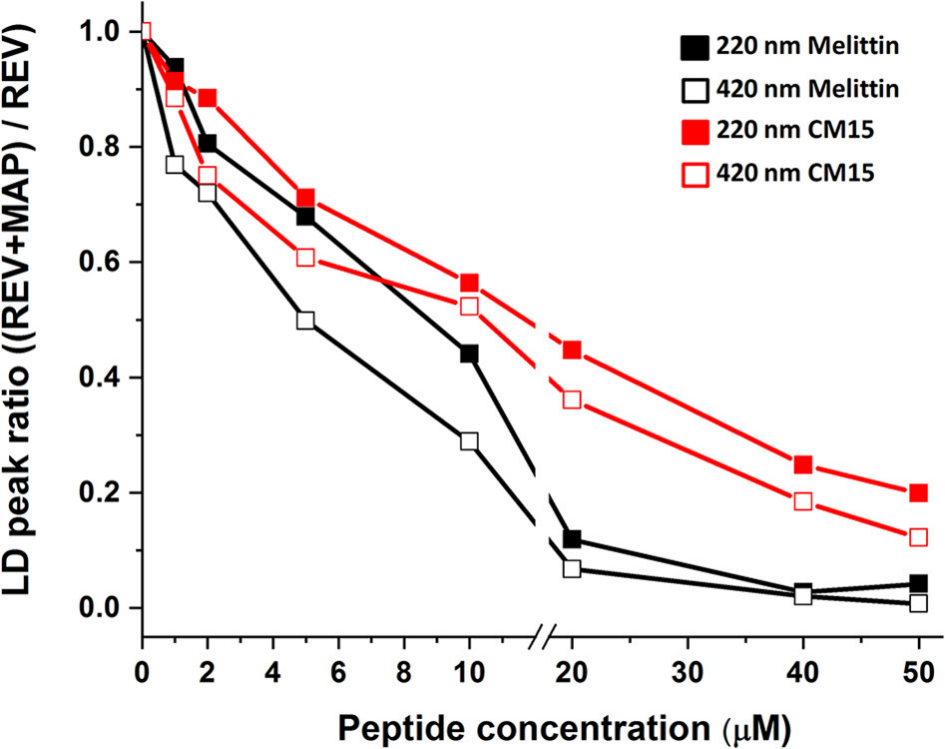
LD signal ratio changes in the presence of peptides. Normalized maximal absorbance intensities of LD peaks at 220 (close square) and 420 nm (open square) in the function of melittin (black) and CM15 (red). The error bars are below 1%.

When comparing signal changes of the LD peak at ~220 nm, arising from amide regions of all oriented proteins, a reduced signal is observed upon increasing peptide concentration (Figures 1, 2). This indicates that MAPs affect all membrane associated proteins involving both membrane embedded or surface associated molecules during the titration process. However, the same trend is found for the Soret band of hemoglobin suggesting its effective removal from the REV surface initiated by these peptides. Further on, the relative loss of peak intensities at 220 and 420 nm during titration follow a similar trend for each particular peptide (Figure 2 and Table S1). Note that while for melittin the signal is almost completely lost at 20 μM peptide concentration, for CM15 the peaks are still observable even at 40 μM suggesting a slower effect for the latter membrane active peptide.

**Figure 3:**
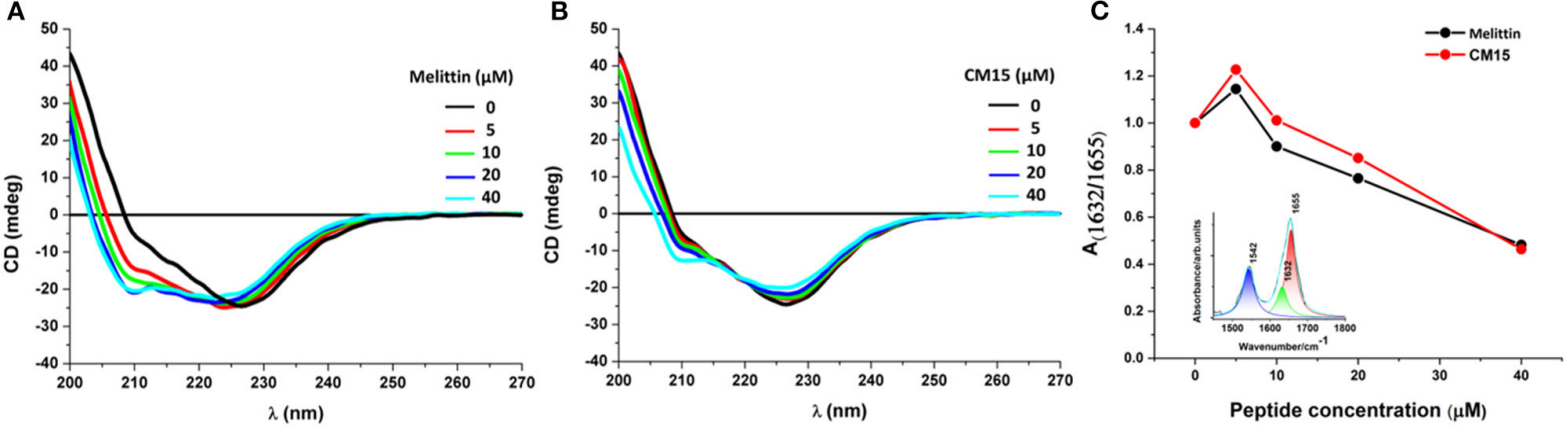
REVs—membrane active peptide interactions studied by CD and IR spectroscopy. Far-UV CD spectroscopic changes of REV samples upon addition of increasing peptide concentrations for melittin **(A)**, and for CM15 **(B)**. Areas ratio **(C)** of amide I components centered at 1632 and 1655 cm^−1^ representing β-sheet and α-helical content, respectively. Inset: curve fitted, deconvoluted REV spectrum (cyan line) in the range 1450–1800 cm^−1^ with three Lorentian curves, corresponding to amide II (blue), β-sheet (green) and α-helix (red) components.

As previously reported (Szigyártó et al., 2018) the CD spectrum of REVs arising from all proteins accompanied with the vesicles exhibits a strong, broad negative band ~226 nm originated from n–π^*^ transition and a much weaker unresolved π−π^*^ transition component at 210 nm. The interactions of MAPs with REVs were investigated using the same titrations as described in the LD section. Addition of melittin and CM15 rendered the decrease of the main negative band at 226 nm (Figures 3A,B) accompanied with small blue shifts. Concomitantly, at higher peptide concentrations, the band at 208 nm displayed as a shoulder was blue shifted and appeared as a separate peak with increased intensity. All of these changes are most prominent for the use of melittin. The spectral variations resemble to those observed previously for REVs upon storage (Szigyártó et al., 2018), where conformational changes of membrane-associated proteins, especially, hemoglobin, have been detected.

To better understand the effect of the membrane-peptide interaction on the global CD spectra, reference measurements using the same titration were also performed with DOPC (dioleoyl-phosphatidylcholine) and DOPC/DOPG (dioleoyl-phosphatidylcholine/dioleyl-phosphatidylglycerol, 80/20 n/n%) liposomes, which can be considered as the simplest model system to mimic the outer leaflet of red blood cell membranes (Viswanad et al., 2014) (Figures S1, S2). When comparing the arithmetic sums of the individual CD curves of the free REV plus peptides (Figure S3) as well as MAPs bound to liposomes (Figure S1) it is obvious that the CD profile found for the REV plus MAP mixtures is distinct from such a simple combination of the two separate components. Nonetheless qualitative similarities can be observed, especially in the increasing helicity upon peptide additions. Nevertheless, the more than 80 proteins that have been identified in REVs (Prudent et al., 2018), are highly likely to undergo some conformational changes especially if they are detached from the surface upon peptides additions, thus the observed CD signal variations cannot be deduced with confidence.

To support changes in secondary structure observed by CD, FTIR spectra were collected focusing on amide I (~1660 cm^−1^) and amide II (~1542 cm^−1^) spectral contributions of proteins and peptides (Barth, 2007). Analysis of the two main band components of amide I, centered at 1632 and 1655 cm^−1^ are characteristic of antiparallel β-sheets and α-helices, respectively (Barth, 2007; Mihály et al., 2017) (Figure 3C). A definite change in the ratio of the two amide I components was observed for both MAPs with increasing concentration, suggesting the dominance of α-helical conformation. This effect can be related to the interaction of the peptides with the REV membrane proteins and with the lipid bilayer. Note, that sample drying required for the IR measurements may influence the structural properties of peptides. However, for both studied systems the results are in close agreement with those of the solution phase CD data, which strongly suggests that such an effect was not significant here.

**Figure 4:**
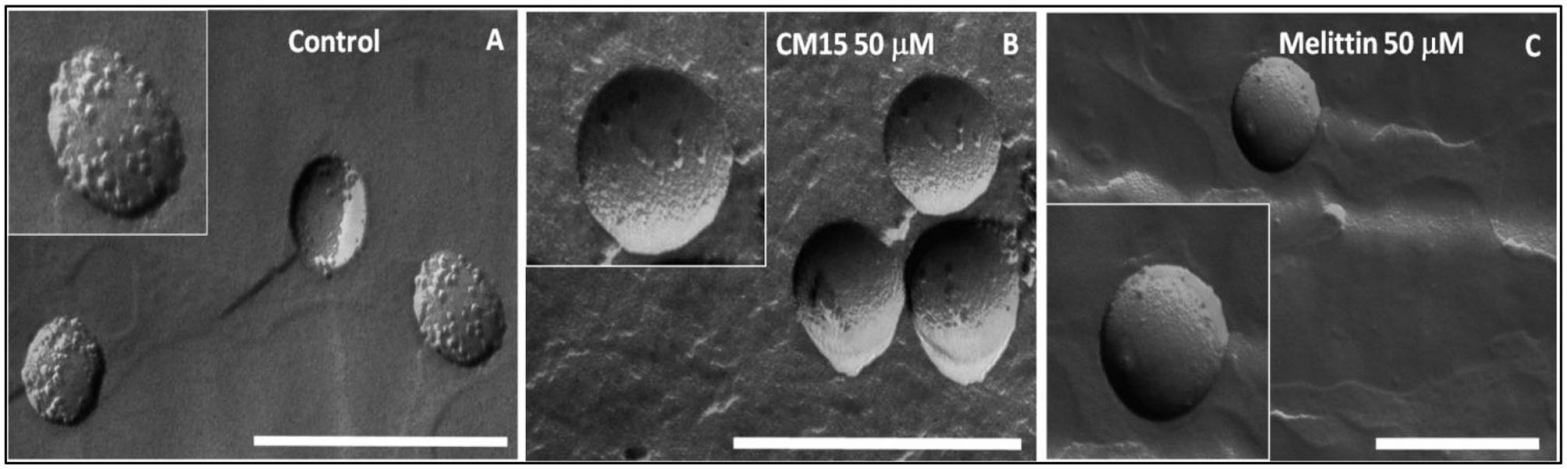
Morphology changes of REV after the addition of MAPs studied by freeze-fracture transmission electron microscopy. FF-TEM images of REV control **(A)** and REV+peptides [peptide concentration 50 μM, CM15 **(B)** and melittin **(C)**] (Scale bar 500 nm).

Qualitative changes on REV morphology was further investigated by transmission electron microscopy. The original REV surface contained granules representing native assemblies of membrane associated proteins (Figure 4A) (Varga et al., 2014, 2020; Deák et al., 2015; Szigyártó et al., 2018). The average size of these vesicles was also verified using dynamic light scattering (DLS) measurements. These measurements indicated that original REVs have spherical morphology with a mean diameter of ~200 nm, which size remains mostly similar after addition of peptides (Table S2 and Figure S4), whereas remarkable changes were observed in REV membrane surface morphology after addition of CM15 and melittin (Figures 4B,C).

Protein aggregation on the vesicle surface and partial removal of surface proteins can be observed already at 5 μM concentration of the MAPs (Figures S5, S6). Further increase in the peptide concentrations up to 50 μM shows significant decrease in the native protein particles by removing them from the vesicle membrane surface (Figures 4B,C). The FF-TEM measurements reveal that both peptides cause detachment of proteins from membrane surface, whereas the membrane remained mainly intact. Proteins detached from the surface can be observed close to the remaining clear intact REV vesicles (Figure S6).

**Figure 5:**
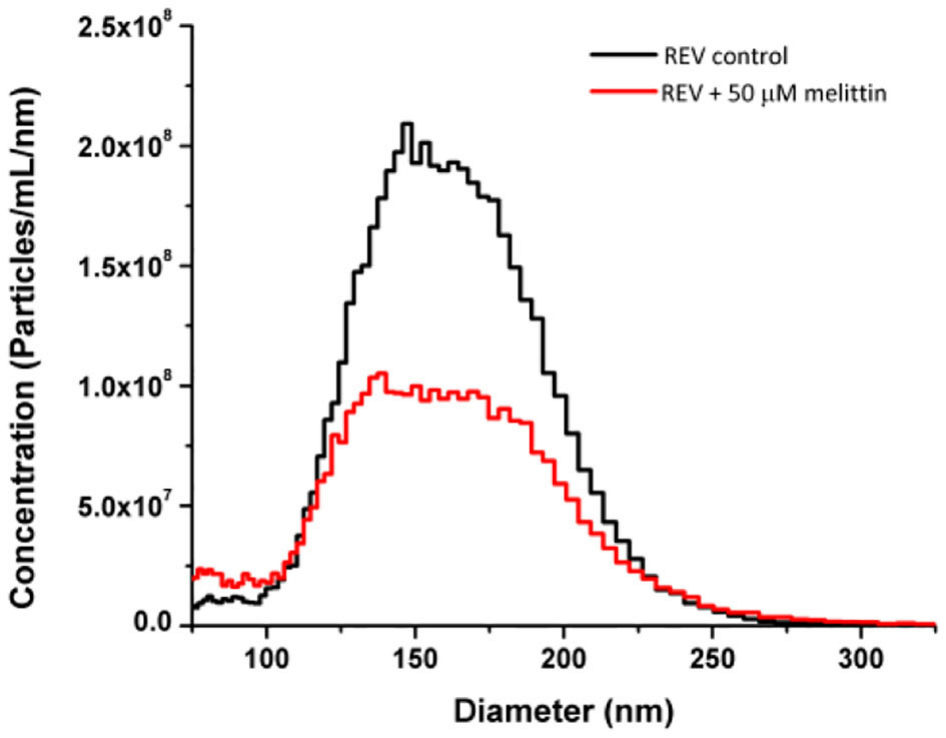
Size distribution and concentration of control REV and REV with melittin samples measured by MRPS. The decrease of the peak corresponds to ~37% loss of particles after the addition of melittin. Peptide concentration was 50 μM.

Note, that melittin is also known to be hemolytic (Sato and Feix, 2006). This could affect the above results as for instance loss of LD signal intensities can be attributed also to membrane disruption. Consequently, to address and quantify how prominent is the membrane disrupting effect of melittin on REVs, microfluidic resistive pulse sensing (MRPS) measurements were employed by determining the size distribution and concentration of vesicles. The absolute size distribution of REVs was determined with and without melittin (Figure 5). The overall particle concentration of the control REV sample was 1.57·10^10^ ± 8·10^7^ particles/mL, which is reduced to 9.58·10^9^ ± 5·10^7^ particles/mL in the presence of 50 μM melittin. This indicates that by the final concentration of our titrations melittin disrupts ~37% of the vesicles. This is in line with the previously described effect of this peptide on red blood cells (Watala and Gwozdzinski, 1992), and is reasonably a low disrupting effect for REVs if one considers that in the samples the used final peptide concentration, 50 μM, corresponds to ~2·10^6^ melittin molecules per one vesicle.

**Figure 6:**
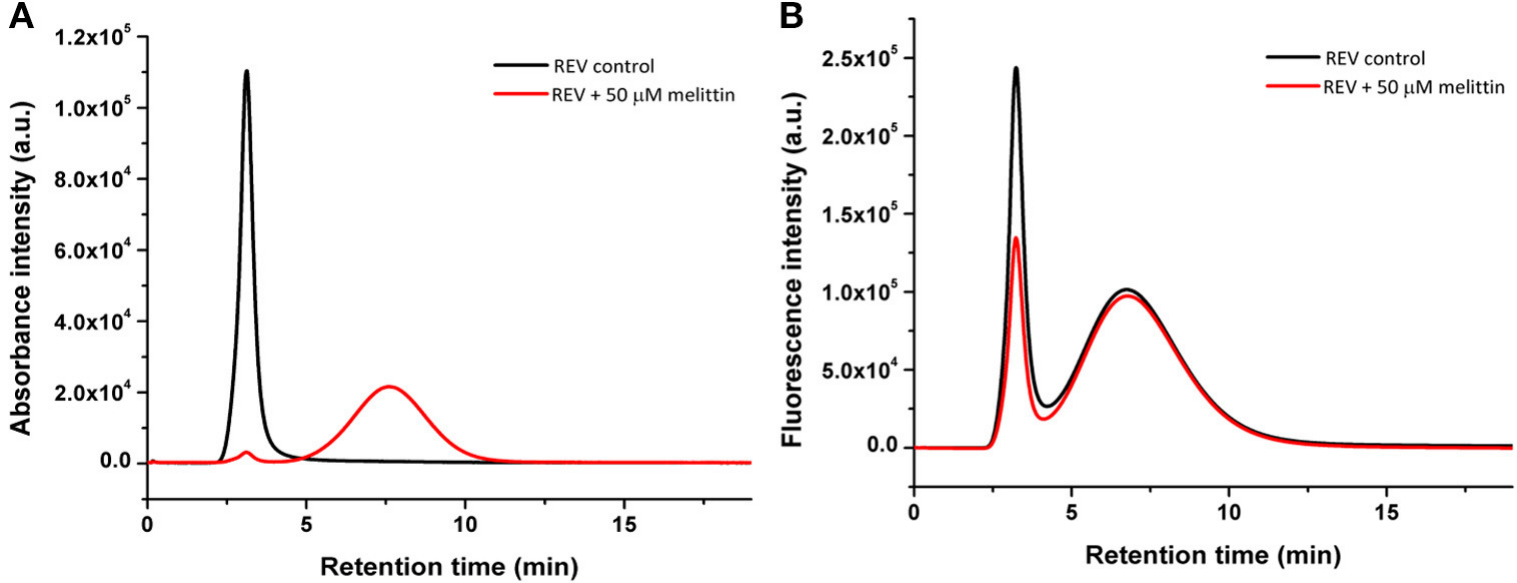
Absorbance at 414 nm **(A)** and fluorescence **(B)** chromatograms of the REV samples without and with melittin. The first peak at around 3 min retention time corresponds to REVs, while the second peak at 7.6 min (absorbance) and 6.8 min (fluorescence) corresponds to free proteins and free PE-anti-CD235a molecules, respectively.

To support MRPS results, and to better compare and understand at a near quantitative level the effect of melittin on both protein corona members and membrane inserted proteins, size exclusion chromatography with fluorescence detection (Flu-SEC) was used. For investigating membrane protein-melittin interactions glycophorin A (CD235a), a characteristic REV membrane protein, was chosen. Control and melittin-treated REVs were labeled with anti-CD235a conjugated with phycoerythrin (PE-CD235a) and the absorbance of hemoglobin as well as the fluorescence of the antibody was detected (Figure 6). The absorbance chromatogram of control REVs shows a single peak at 3.1 min retention time, which corresponds to the void volume of the column, and indicates intact EVs (Figure 6A). In contrast, for REVs treated with 50 μM melittin the peak was remarkably reduced in intensity with only 3% area (AUC) compared to the control sample, and a new peak appears at 7.6 min retention time, which corresponds to hemoglobin molecules detached from REVs.

Monitoring the fluorescence chromatogram of control REV (Figure 6B) two peaks at 3.2 and 6.5 min retention times corresponding to REV-bound and free antibodies were observed. The intensity of the REV peak at 3.2 min is reduced to 55.9% in the presence of melittin compared to the control sample. This reduction is in good agreement with the loss of vesicle number determined by MRPS measurements, i.e., the reduced REV-peak on the fluorescence chromatogram can be attributed to the decreased REV concentration. These observations drive to the conclusion that melittin is capable to remove surface bound proteins but does not affect significantly the membrane-embedded molecules like glycophorin A. The loss of signal intensity lies close to that observed for MRPS measurements indicating a fraction of the vesicles being disrupted.

The combined results confirm that both melittin and CM15 efficiently remove proteins adsorbed to the external surface of REVs, which is a new functionality that has not been seen for these relatively well-studied MAPs. The TEM images clearly display that the REV surfaces will become much smoother. As hemoglobins is one of the most abundant proteins found in human protein corona (Solorio-Rodríguez et al., 2017) in line with this, the abrupt decrease of the Soret band at 420 nm indicates the removal of membrane-bound, LD active hemoglobin molecules. Furthermore, since melittin can form membrane pores (Smith et al., 1994; Hur et al., 2017) it is likely that besides removing hemoglobins from the external surface, it also causes leakage of internal hemoglobins from the vesicles. This assumption is supported by Flu-SEC results of REVs treated with melittin showing almost complete loss of peak intensity of REV-associated hemoglobin (Figure 6A). The TEM images show that the proteins removed from the surface aggregate most likely together with the peptides, and their assemblies can be found quite near to smooth vesicles from which they are likely to directly originate. Based on the MRPS and fluorescence-SEC measurements membrane inserted proteins most likely are only little affected. This is also supported by LD spectra collected at higher melittin concentrations, where the peak ~220 nm gradually disappears, and a smaller negative band appears at 208 nm. While the initial peak will be a contribution from all the proteins present on the oriented vesicles, and thus challenging to interpret, the latter negative peak will likely arise from a narrower distribution of structural elements. Its absolute value lies close to helices typically inserted into the bilayer preferentially parallel to the membrane normal, as this band arises from the π−π^*^ amide transitions that are polarized along the helix axis (Figure 1A) (Nordén et al., 2010).

## Conclusions

In summary, current results demonstrate that melittin and CM15 drastically affects protein corona adsorbed to the REV lipid bilayer. The detachment of these surface proteins by these MAPs can clearly be reached and tracked, potentially providing improved applications for surface engineering and production of EV-based therapeutics enhancing particle-based delivery strategies (Bhunia et al., 2017; Nguyen and Lee, 2017). Increased understanding on these interactions and how this can be controlled by other biomolecules is an interesting direction, which we aim to explore in the future. The combination of several biophysical techniques also allowed the qualitative separation and the nearly quantitative identification of apparent lysis and surface protein-removing activities of melittin, which offers improved understanding for different processes occurring simultaneously during peptide-biomembrane interactions. The observed new functionality of the studied peptides suggests that they may have further alternative activities *in vivo* beyond those observed previously on synthetic liposomes. Results also imply that extracellular vesicles provide an excellent opportunity to better follow and understand interactions of membrane active compounds with complex biomembranes. Considering that membrane active peptides are also interesting for loading various drug compounds into EVs and for aiding their trans-bilayer penetration, we expect that peptide-EV interactions will receive increasing focus in EV-engineering.

## Data Availability Statement

The raw data supporting the conclusions of this article will be made available by the authors, without undue reservation.

## Ethics Statement

The studies involving human participants were reviewed and approved by Scientific and Research Ethics Committee of the Hungarian Medical Research Council (ETT TUKEB 6449-2/2015). The patients/participants provided their written informed consent to participate in this study.

## Author Contributions

TB-S, IS, and ZV conceived the study. PS and IS performed CD, LD, ATR-FTIR experiments, and data analysis. MR and JM contributed to ATR-IR data evaluation and discussion. FZ and TJ contributed to data interpretation and discussion. SB performed peptide synthesis. ÉB and JK performed FF-TEM measurements. DK and ZV performed MRPS and Flu-SEC experiments and data evaluation. IS and TB-S wrote the manuscript. All authors contributed to the article and approved the submitted version.

## Conflict of Interest

The authors declare that the research was conducted in the absence of any commercial or financial relationships that could be construed as a potential conflict of interest.

## Supplementary Material

Experimental procedures and data: liposomes preparation, CD, DLS, TEM measurements.
