# Supplementary material for: Membrane Active Peptides Remove Surface Adsorbed Protein Corona From Extracellular Vesicles of Red Blood Cells
Source: Front Chem. 2020 Aug 11;8:703. doi: 10.3389/fchem.2020.00703 (PMC7432246; doi:10.3389/fchem.2020.00703)
Supplement: Supplementary file 1 [file Data_Sheet_1.docx]

Supplementary Material

***Chemicals***

Physiological salt solution (9 g NaCl in 991 g Millipore water), isotonic phosphate buffered saline (PBS, 10 mM phosphate, 137 mM NaCl, 3 mM KCl, pH 7.4) were purchased from Sigma-Aldrich. Bovine serum albumin standard was supplied by Bio-Rad Hungary Ltd. Melittin was purchased from NovoPro BioScience. CM-15 was provided by the Research Group of Peptide Chemistry, MTA-ELTE ^1^. Peptide content was determined by amino acid analysis; CM-15: 52% and melittin: 57%. 1,2-Dioleoyl-*sn*-glycero-3-phosphocholine (DOPC) and 1,2-dioleoyl-sn-glycero-3-[phospho-rac-(1-glycerol)], sodium salt (DOPG) was purchased from Avanti Polar Lipids Inc. (USA). The use of human blood samples was approved by the Scientific and Research Ethics Committee of the Hungarian Medical Research Council (ETT TUKEB 6449-2/2015) and all procedures were performed in agreement with the declaration of Helsinki. Red blood cells were isolated from the blood of healthy volunteers, collected at the Central Laboratory of the National Institute of Rheumatology and Physiotherapy, Budapest, Hungary. Anti-CD235a (anti - Glycophorin A) conjugated with phycoerythrin (PE-CD235a) was purchased from BioLegend, USA

***DOPC and DOPC/DOPG Liposome***

DOPC and DOPC/DOPG (molar ratio 80/20) liposomes with a diameter around 200 nm and at a lipid concentration of 12.7 mM were prepared by the extrusion method. Briefly, lipid was dissolved in chloroform containing 50 vol % methanol, which was then evaporated. The lipid film obtained was kept in vacuum for 8 hours to remove the residual traces of solvent. The dried lipid film was then hydrated with PBS buffer. To achieve homogenous mixture, repeated heating (37ºC) and cooling (-196ºC) steps were applied. The solution was extruded through polycarbonate filters with 200 nm pore size (at least 10 times) using a LIPEX extruder (Northern Lipids Inc., Canada).

***Linear dichroism spectroscopy***

**Table S1.** Change of surface protein content as a function of MAPs concentrations

| **Peptide concentration (µM)** | Relative values | | | |
| --- | --- | --- | --- | --- |
|  | Melittin ratio  220 nm | CM15 ratio  220 nm | Melittin ratio  420 nm | CM15 ratio  420 nm |
| 0 | 1.00 | 1.00 | 1.00 | 1.00 |
| 1 | 0.94 | 0.91 | 0.77 | 0.88 |
| 2 | 0.81 | 0.88 | 0.72 | 0.75 |
| 5 | 0.68 | 0.71 | 0.50 | 0.61 |
| 10 | 0.44 | 0.56 | 0.29 | 0.52 |
| 20 | 0.12 | 0.45 | 0.07 | 0.36 |
| 40 | 0.03 | 0.25 | 0.02 | 0.18 |
| 50 | 0.04 | 0.19 | 0.01 | 0.12 |

***Circular dichroism spectroscopy***


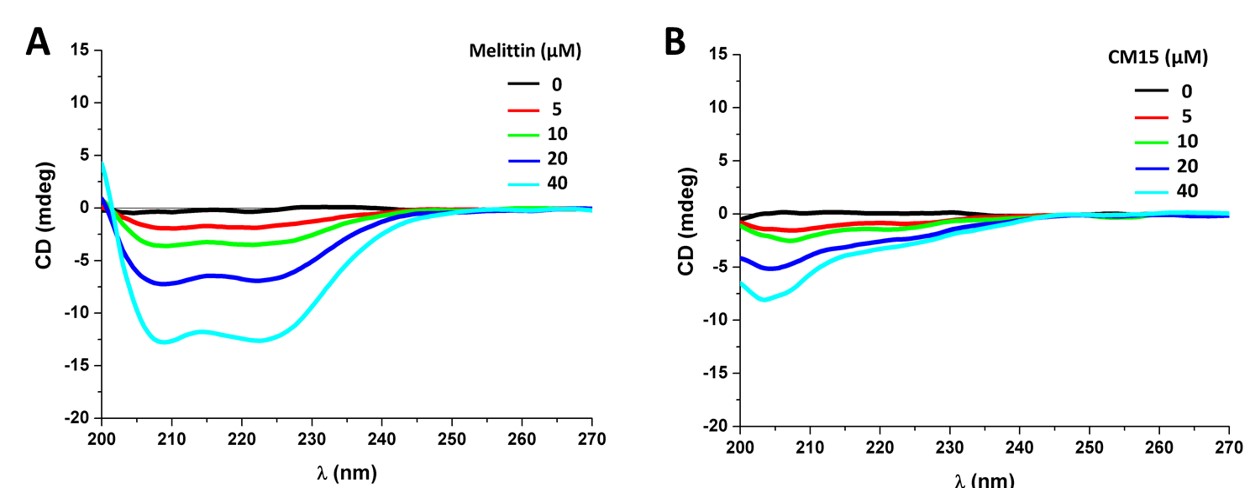


**Figure S1.** DOPC liposome MAPs interactions studied by CD spectroscopy. Far-UV CD spectroscopic changes of DOPC sample measured after consecutive addition of increase peptides concentrations (A (melittin) and B (CM15)). Liposome concentration was 1.27 mM.

**
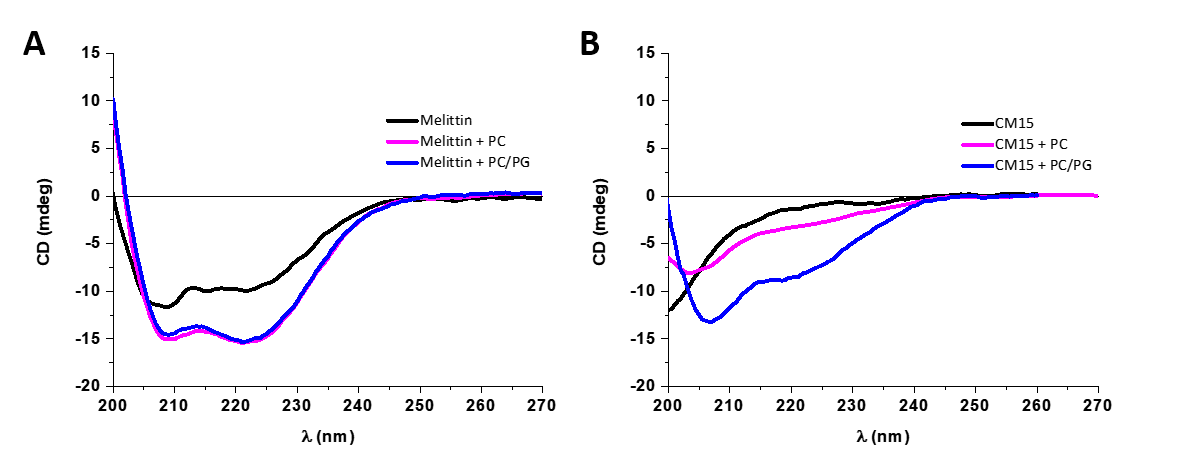
**

**Figure S2.** Far-UV CD spectra (A) melittin and (B) of CM15 (40 µM) in the presence of DOPC (PC) and DOPC/DOPG (PC/PG) liposomes (1.27 mM total lipid).


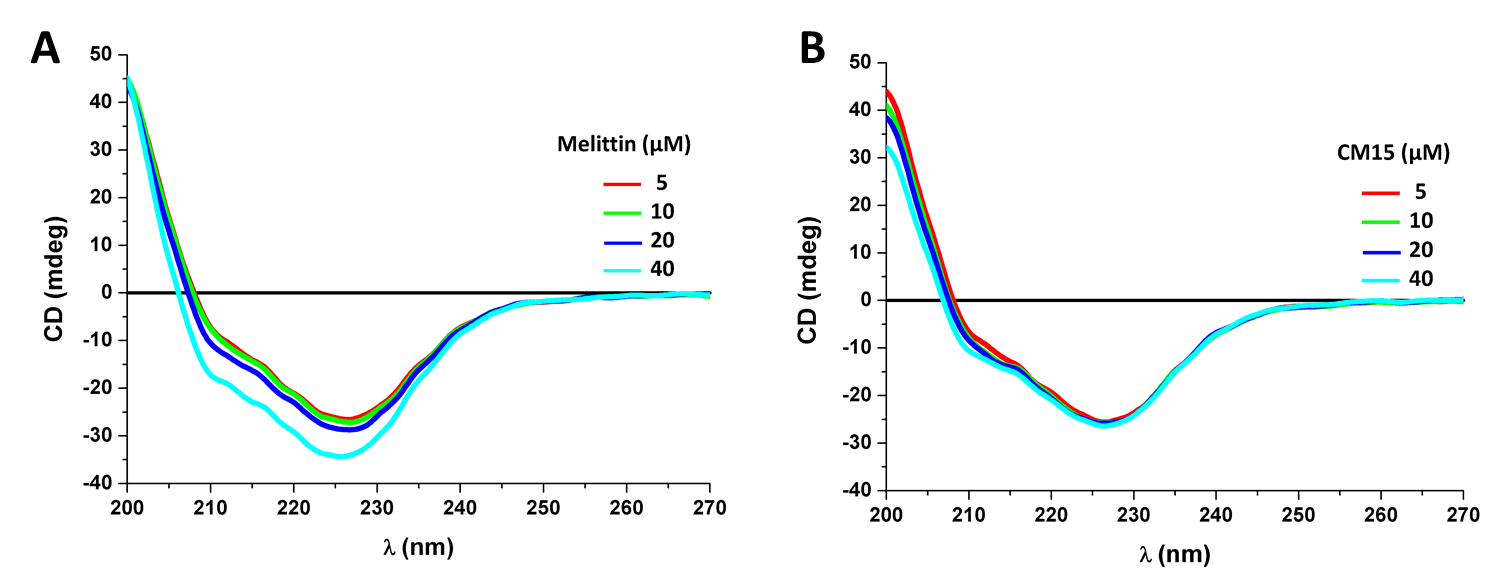


**Figure S3.** Arithmetic sum of the individual CD curves of REV and free MAPs (A (melittin) and B (CM15))

***Dynamic Light Scattering***

**Table S2.** DLS measurements on REV samples after the addition of MAPs

| **Sample** | **Mean diameter (nm)** | **Polydispersity (%)** |
| --- | --- | --- |
| **REV** | 215±25 | 12 |
| **REV + Melittin** |  |  |
| 5 μM | 209±31 | 15 |
| 10 μM | 213±40 | 40 |
| 20 μM | 217±52 | 24 |
| 40 μM | 225±50 | 22 |
| **REV + CM15** |  |  |
| 5 μM | 210±27 | 13 |
| 10 μM | 224±15 | 7 |
| 20 μM | 211±51 | 24 |
| 40 μM | 218±28 | 13 |

**
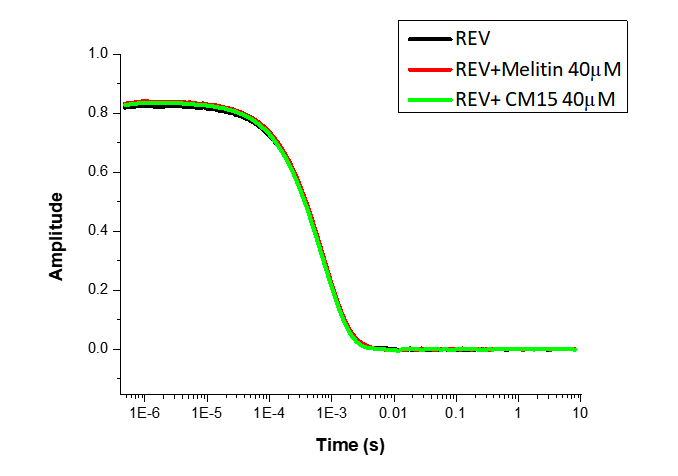
 Figure S4.** Correlation function of particle size measured by DLS for REV sample with successive addition of MAPs

***Freeze-fracture transmission electron microscopy***


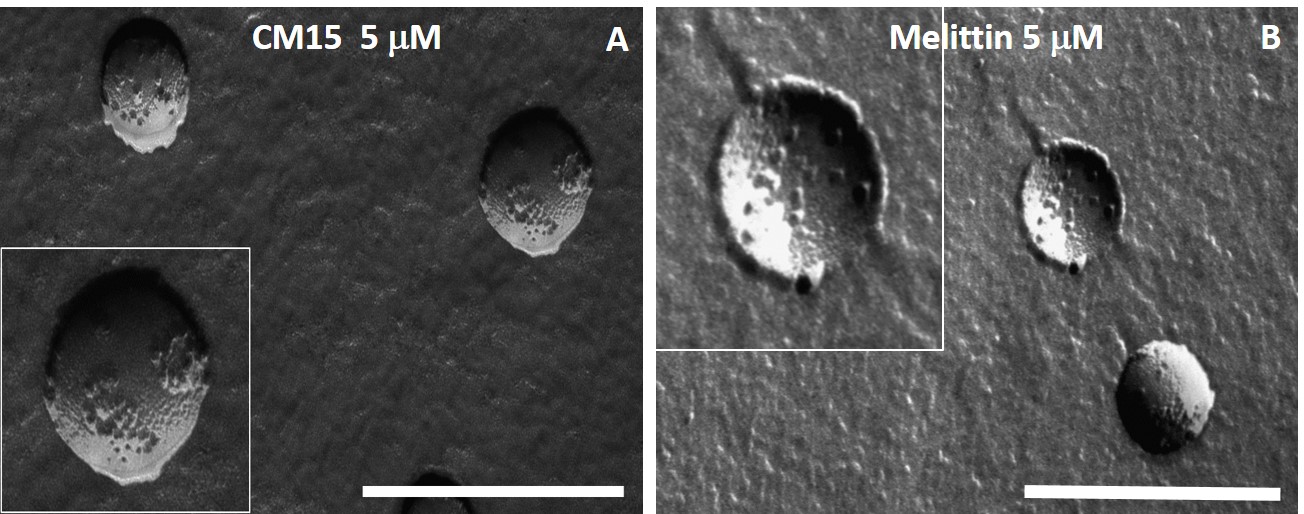
**Figure S5.** FF-TEM image of REV+MAPs measured after addition of 5 µM peptide (CM15 (A) and melittin (B)) (Scale bar 500 nm).

**
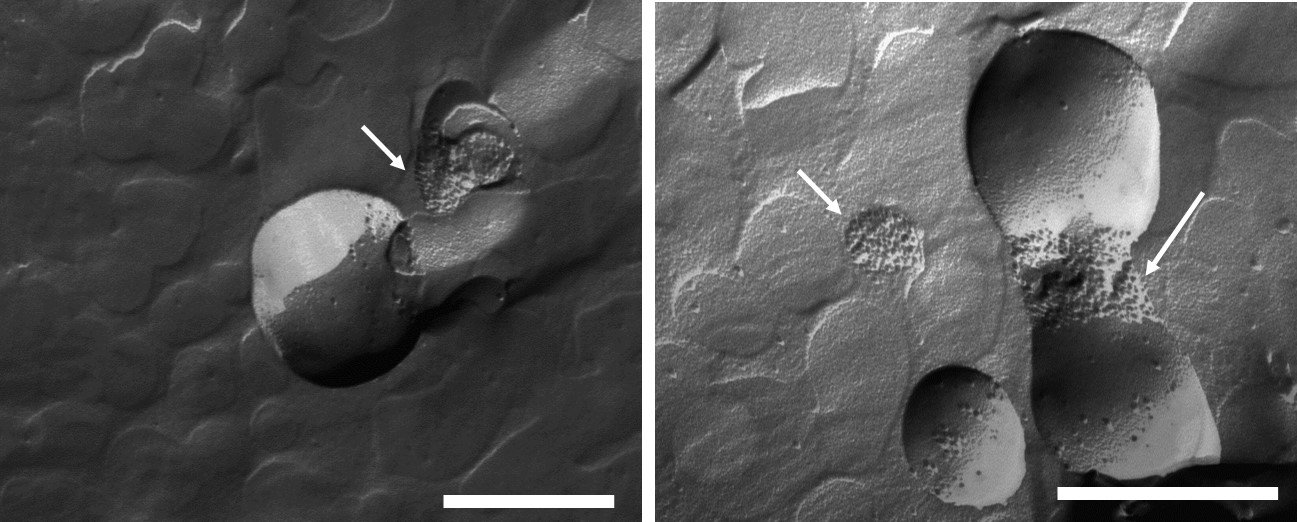
**

**Figure S6.** FF-TEM of REV measured after addition of 50 µM melittin concentration, arrows indicating detached proteins from REV membrane surface in the background (Scale bar 500 nm).

**References**

1. Horváti, K. *et al.* Comparative analysis of internalisation, haemolytic, cytotoxic and antibacterial effect of membrane-active cationic peptides: aspects of experimental setup. *Amino Acids* **49**, 1053–1067 (2017).
